# Class II, Division 1 Angle malocclusion with severe proclination of maxillary incisors

**DOI:** 10.1590/2177-6709.21.1.101-109.bbo

**Published:** 2016

**Authors:** Kátia Montanha

**Affiliations:** 1 Specialist in Orthodontics, Pontifícia Universidade Católica de Minas Gerais (PUC-MG), Belo Horizonte, Minas Gerais, Brazil

**Keywords:** Class II Angle malocclusion, Orthodontic headgear, Corrective Orthodontics

## Abstract

Protrusion of maxillary incisors is a common complaint among patients seeking orthodontic treatment. This report addresses the correction of Class II Angle malocclusion with excessively bucally proclined maxillary incisors, in an adolescent female patient, through the use of extraoral and fixed appliances. This case was presented to the Brazilian Board of Orthodontics and Dentofacial Orthopedics (BBO) as part of the requirements for obtaining the title of certified by the BBO.

## Abstract (pt)

A protrusão dos incisivos superiores é uma queixa frequente nos pacientes que buscam o tratamento ortodôntico. O presente relato aborda a correção de uma má oclusão de Classe II de Angle, com severa protrusão e inclinação para vestibular dos incisivos superiores, em uma paciente adolescente do sexo feminino, por meio do uso de aparelhagem extrabucal e fixa. Esse caso clínico foi apresentado à Diretoria do Board Brasileiro de Ortodontia e Ortopedia Facial (BBO), como parte dos requisitos para a obtenção do título de Diplomada pelo BBO.

## INTRODUCTION

An 11-year-old, Caucasian, female patient was referred by her parents for orthodontic evaluation, and had the main complaint of having her teeth "sticking out." She presented with good general health upon initial examination. During anamnesis, no major medical record was found, and her parents revealed she had the nocturnal habit of teeth grinding and naso-oral breathing pattern. Clinical examination revealed no dental wear, nor changes in tongue posture at rest or in function. The opening and closing movements of the jaw showed normal range, with no deviation and no noise, and the permanent dentition was complete, with the maxillary right canine almost fully erupted.

## DIAGNOSIS

**Figure 1:**
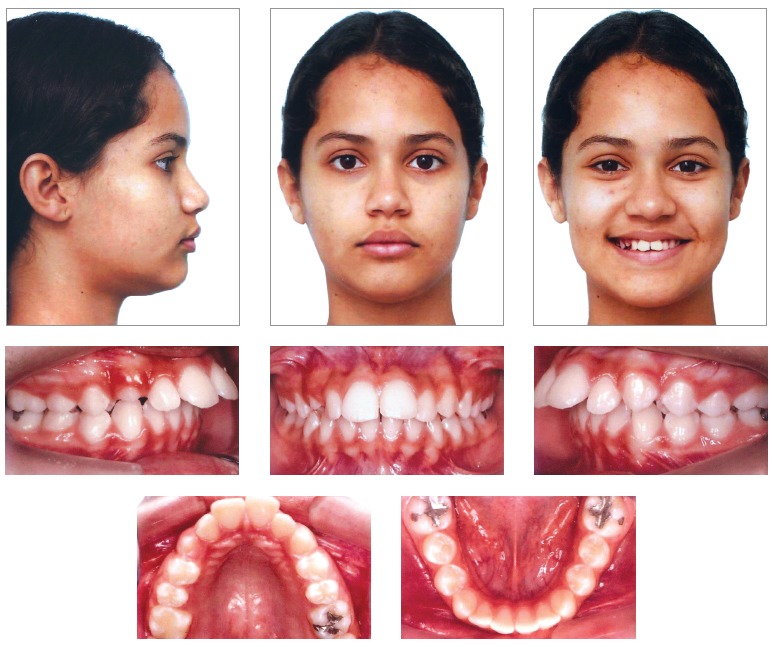
- Initial facial and intraoral photographs.

Facial analysis in frontal view revealed passive lip seal, adequate exposure of incisors with lips at rest, the upper midline coinciding with the facial median sagittal plane, and normal smile line. Analysis in lateral view revealed a convex profile, with prominent upper lip, nasolabial and lip-chin angle of 90°, and slightly decreased lower facial height. These aspects can be seen in Figure 1.

**Figure 2:**
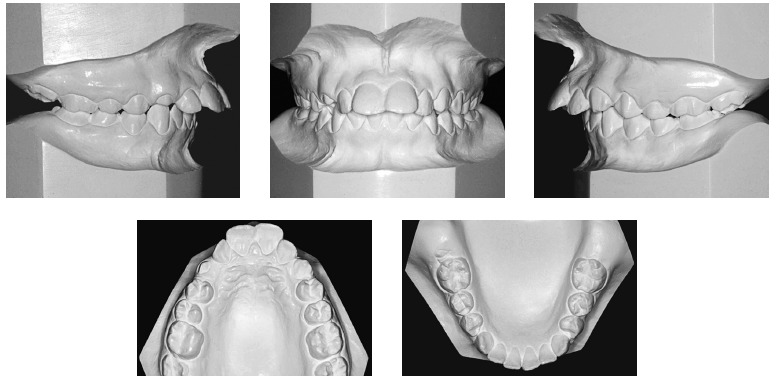
- Initial casts.

The intraoral evaluation (Figs 1, 2) revealed Class II, Division 1 malocclusion, severe overbite, mandibular incisors touching the palatal mucosa, severe overjet of 10.5 mm, accentuated curve of Spee and coinciding upper and lower midlines.

**Figure 3:**
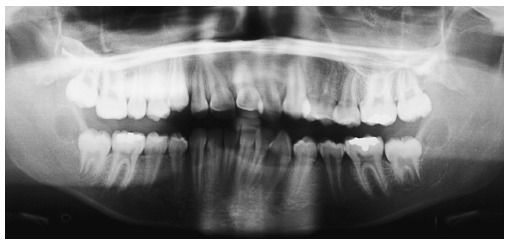
- Initial panoramic radiograph.

Panoramic radiograph (Fig 3) revealed the appropriate process of root formation of permanent teeth, and the presence of third molars, in the early stages of formation.

**Figure 4:**
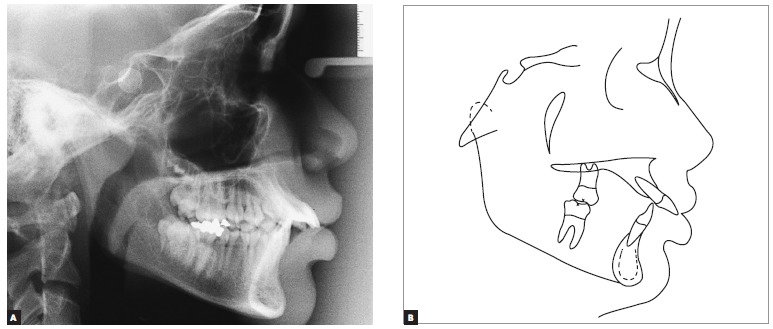
- Initial cephalometric radiograph (A) and tracing (B).

Cephalometric analysis (Fig 4) revealed a skeletal Class I pattern (ANB = 2° and Wits = 0 mm) and a slightly decreased lower third of the face (SN-GoGn = 30° and FMA = 22°). Mandibular incisors were well positioned (1-NB = 25° and 4 mm), while maxillary incisors were severely proclined and protruded (1-NA = 52° and 15 mm), being responsible for the decrease in the interincisal angle (1/1 = 102°). These and other cephalometric values ​​are presented in Table 1.

**Table 1:** - Initial (A) final (B) cephalometric values.

	**Measurements**		**Normal**	**A**	**B**	**Dif. A/B**
Skeletal pattern	SNA	(Steiner)	82°	78°	79°	1
	SNB	(Steiner)	80°	76°	77°	1
	ANB	(Steiner)	2°	2°	2°	0
	Wits	(Jacobson)	♀ 0 ± 2 mm ♂ 1 ± 2 mm	0 mm	2 mm	2
	Angle of convexity	(Downs)	0°	0°	0°	0
	Y-axis	(Downs)	59°	56°	54°	2
	Facial angle	(Downs)	87°	88°	92°	4
	SN-GoGn	(Steiner)	32°	30°	32°	2
	FMA	(Tweed)	25°	22°	20°	2
Dental pattern	IMPA	(Tweed)	90°	97°	99°	2
	1.NA (degrees)	(Steiner)	22°	52°	24°	28
	1-NA (mm)	(Steiner)	4 mm	15 mm	7 mm	8
	1.NB (degrees)	(Steiner)	25°	25°	27°	2
	1-NB (mm)	(Steiner)	4 mm	4 mm	7 mm	3
	- Interincisal angle	(Downs)	130°	102°	125°	23
	1-APo	(Ricketts)	1 mm	15 mm	6 mm	9
Profile	Upper lip - S-line	(Steiner)	0 mm	2 mm	0.5 mm	1,5
	Lower lip - S-line	(Steiner)	0 mm	4 mm	4 mm	0

## TREATMENT PLAN

Given this situation, for Class II molar relationship correction, treatment plan included the use of Kloehn headgear appliance (KHGA). Subsequently, an orthodontic fixed appliance would be used to distalize maxillary premolars and canines, and retract maxillary incisors. The extraoral appliance was maintained as an anchorage unit, along with a tie together of maxillary posterior teeth.^1^^-^ 6 A sequence of 0.014-in and 0.016-in nickel titanium and 0.018-in and 0.020-in stainless steel wires with stops would be used for levelling and alignment. For maxillary incisors retraction, it should be used a TMA 0.019 x 0.025-in arch wire with T-loops and accentuated curve of Spee.

In the lower arch, the fixed appliance would aim to promote proper alignment, levelling of the curve of Spee and coordination with the upper arch, following a similar sequence of arch wires used in the upper arch.

As for finishing procedures, the chosen arches should be made from 0.018 x 0.025-in and 0.019 x 0.025-in stainless steel wires in the upper and lower arches, with first and third orders individual bends, as necessary. Also if necessary, intermaxillary Class II and/or interdigitation elastics were prescribed.

In the retention stage of treatment, planning included, for the upper arch, a removable wraparound retainer, with a recommended use of 22 hours per day for 12 months, followed by night time use for another 12 months. For the lower arch, a fixed, bonded canine-to-canine retainer, made from 0.020-in stainless steel wire was prescribed. It was also planned to request extraction of third molars.

## TREATMENT PROGRESS

**Figure 5:**
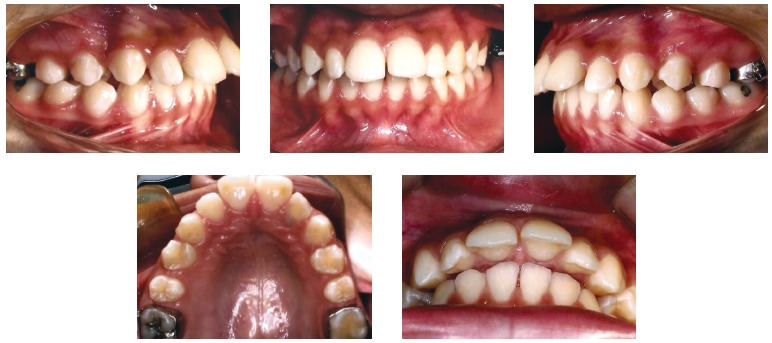
- Intermediate intraoral view, after maxillary molars distalization with the Kloehn headgear.

As planned, headgear tubes were soldered on orthodontic bands, adapted in the maxillary first permanent molars, and the Kloehn headgear appliance (KHGA) was installed with a magnitude of 500 gF force. The patient was advised to use the KHGA for a minimum of 14 hours a day, but encouraged to make use of it for as long as possible. After six months of proper use, the maxillary molars were in a Class I relationship, with generalized spaces in the posterior region, especially in between premolars (Fig 5). Seven months after exclusive use of the headgear, the patient underwent fixed orthodontic mechanotherapy with Roth prescription metallic brackets (0.022 x 0.028-in slot) in the upper arch, including second molars. A sequence of 0.014-in and 0.016-in nickel titanium and 0.018-in and a 0.020-in stainless steel wires with stops were used. Posterior teeth were laced in tie together to reinforce anchorage, and Kloehn appliance use was maintained during the night. Premolars and canines distalization was then started. Periapical radiographs of incisors were obtained after six months of fixed appliance use. When canine Class I relationship was obtained, incisors retraction started with a TMA 0.019 x 0.025-in arch wire with T-loops and accentuated curve of Spee.

The fixed appliance was installed in the mandible eight months later, with the same archwire sequence adopted for levelling and alignment. First and third order bends were added, including intrusion step bends in the mandibular incisors. Six months after the installation of the fixed appliance in the lower arch, new periapical radiographs were requested.

After maxillary incisors retraction was completed, a panoramic radiograph was requested to evaluate root parallelism. Some brackets were repositioned, and new arches made ​​from 0.018 x 0,025-in and 0.019 x 0,025-in stainless steel wire were placed in the upper and lower arches. For canines and first premolars, intermaxillary Class II elastics (1/4-in, 300 gF) in both arches, followed by interdigitation elastics (3/16-in, 250 gF) were recommended. The functional occlusal patterns were tested, and with excursive movements of protrusion, right and left lateral guidance being obtained without interference, the fixed appliance was removed from both arches.

For the retention phase, the patient was instructed to wear a wraparound removable retainer in the upper arch, 22 hours per day. In the lower arch, a fixed retainer made from a 0.020-in stainless steel wire was bonded canine-to-canine. After evaluation of the final records, extraction of third maxillary and mandibular molars was requested.

## RESULTS

**Figure 6:**
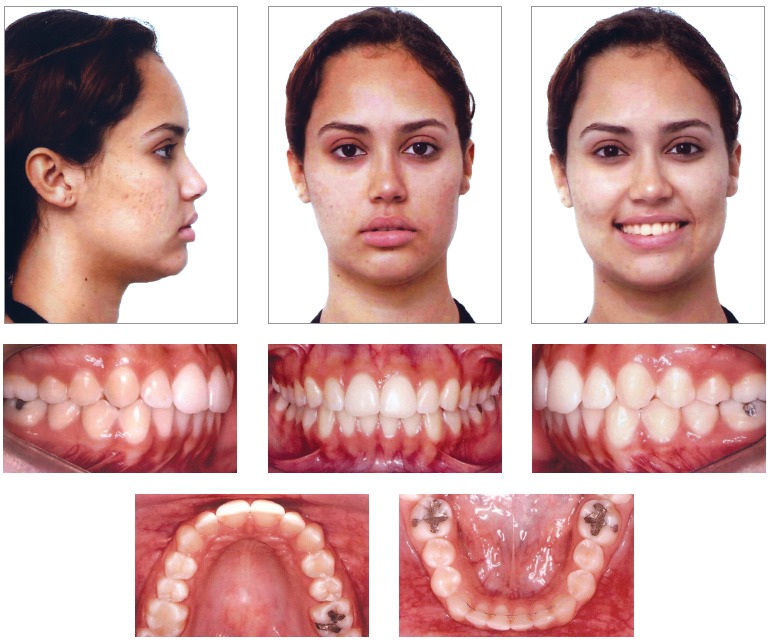
- Final facial and intraoral photographs.

**Figure 7:**
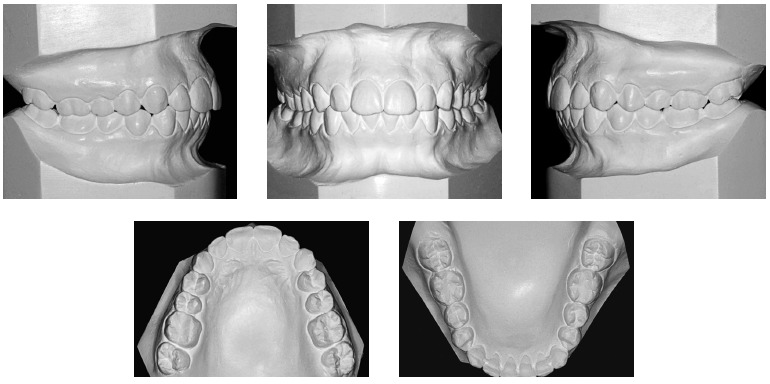
- Final casts.

**Figure 8:**
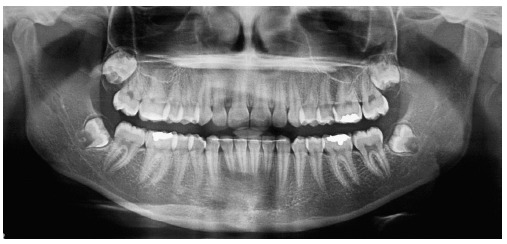
- Final panoramic radiograph.

**Figure 9:**
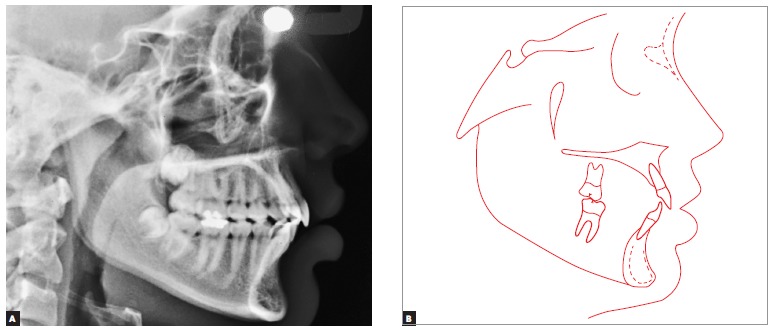
- Final cephalometric radiograph (A) and tracing (B).

Upon evaluation of the final records of the patient (Figs 6 to 9, Table 1), all objectives initially intended appeared to have been achieved, with satisfactory facial and occlusal outcomes. There was significant improvement of facial profile, with retraction of the upper lip, which went from a 2-mm position in relation to the S-line (Steiner) to 0.5 mm. This improvement was due to the significant retraction of upper incisors which were reposition in the maxillary bone, with the 1-NA angle modified from 52° to 24°, and its linear position decreased in 8 mm, from 15 mm to 7 mm.

Class II relationship was corrected, and molar and canine Class I relationships were achieved, with appropriate overbite and overjet obtained. The final incisor position also allowed a mutually protected occlusion, with excursive movements of protrusion and right and left lateral guidance achieved without interference and with canine guidance.

In the mandible, there was levelling of the curve of Spee, which contributed to reduce overbite. There was little change in anteroposterior positioning of incisors, with a slight increase in inclination (1-NB and IMPA angles suffered an increase of 2°, rising from 25° to 27°, and from 95° to 97°, respectively) and linear positioning (1-NB changed from 4 mm to 7 mm). This change was probably due to the use of intermaxillary Class II elastics, required to obtain proper Class I relationship during the finishing phase.

**Figure 10:**
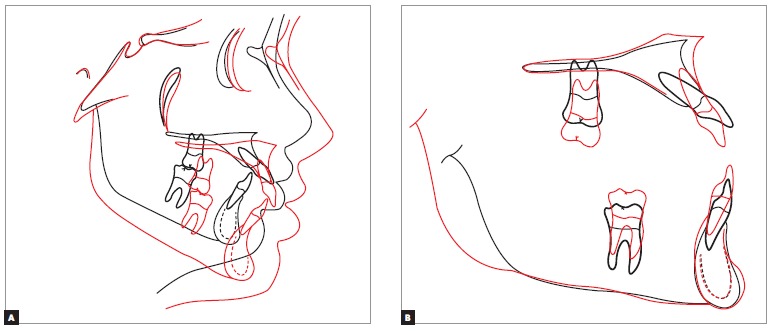
- Total (A) and partial (B) superimpositions. Initial (black) and final (red).

Cephalometric analysis (Fig 10) revealed, in total superimposition, that facial growth was manifested in down and forward direction, and that the ANB angle of 2° did not change, thus maintaining skeletal harmony and achieving correction of facial convexity. A significant improvement in overjet and overbite can also be observed, with profile showing an improvement in lower lip eversion. Partial superimposition of the maxilla reveals alveolar growth in the region of molars and incisors, following the growth of the midface, and a significant change in incisors proclination. In the mandible, there was alveolar growth of the anterior and posterior regions, in addition to proper torque control, thus keeping mandibular incisors inclination.

## FINAL CONSIDERATIONS

Since the end of the last century, many devices have been described as alternatives to the use of extraoral appliance for distal movement of maxillary molars.^2^^,^ 4 ^-^ 10 Most of the times, these new devices have the major advantage of not depending on patient's cooperation. In spite of this, the extraoral appliance remains a legitimate indication for Class II relationship correction and anchorage control. Provided that it is properly used, it yields effective and stable results.

The treatment reported herein had its success guaranteed by the proper use of KHGA, not only during distalization of molars, but also untill the completion of maxillary incisors retraction. Thus, the molar relationship was corrected, spaces were achieved for premolars and canines distalization and the posterior region was anchored during maxillary incisors retraction, allowing the evolution of the mechanics without losing the progress made in each stage of treatment.

During treatment, the patient was on the onset of puberty, a phase that sometimes is a bit complex regarding self-esteem issues. For this reason, the orientation was to use the extraoral appliance for about 14 hours a day, at night and during the period she was not at school, therefore not interfering in her social life. At the end, the patient and her parents were quite satisfied with the results, regarding both facial and dental aspects.

## References

[1] Proffit WR, Fields HW, Sarver DM (2013). Contemporary Orthodontics.

[2] Miller RA, Tieu L, Flores-Mir C (2013). Incisor inclination changes produced by two compliance-free Class II correction protocols for the treatment of mild to moderate Class II malocclusions. Angle Orthod.

[3] Godt A, Berneburg M, Kalwitzki M, Göz G (2008). Cephalometric analysis of molar and anterior tooth movement during cervical headgear treatment in relation to growth patterns. J Orofac Orthop.

[4] Nanda RS, Dandajena TC (2006). The role of the headgear in growth modification. Semin Orthod.

[5] Kirjavainen M, Hurmerinta K, Kirjavainen T (2007). Facial profile changes in early Class II correction with cervical headgear. Angle Orthod.

[6] Sloss EA, Southard KA, Qian F, Stock SE, Mann KR, Meyer DL (2008). Comparison of soft-tissue profiles after treatment with headgear or Herbst appliance. Am J Orthod Dentofacial Orthop.

[7] Kinzinger GS, Eren M, Diedrich PR (2008). Treatment effects of intraoral appliances with conventional anchorage designs for non-compliance maxillary molar distalization: a literature review. Eur J Orthod.

[8] Sfondrini MF, Cacciafesta V, Sfondrini G (2002). Upper molar distalization: a critical analysis. Orthod Craniofac Res.

[9] Patel MP, Henriques JF, Freitas KM, Grec RH (2014). Cephalometric effects of the Jones Jig appliance followed by fixed appliances in Class II malocclusion treatment. Dental Press J Orthod.

[10] Chhibber A, Upadhyay M, Uribe F, Nanda R (2013). Mechanism of Class II correction in prepubertal and postpubertal patients with Twin Force Bite Corrector. Angle Orthod.

